# Normalized power variance of eLORETA at high-convexity area predicts shunt response in idiopathic normal pressure hydrocephalus

**DOI:** 10.1038/s41598-020-70035-9

**Published:** 2020-08-03

**Authors:** Yasunori Aoki, Hiroaki Kazui, Ricardo Bruña, Roberto D. Pascual-Marqui, Kenji Yoshiyama, Tamiki Wada, Hideki Kanemoto, Yukiko Suzuki, Takashi Suehiro, Takuya Matsumoto, Kyosuke Kakeda, Masahiro Hata, Leonides Canuet, Ryouhei Ishii, Masao Iwase, Manabu Ikeda

**Affiliations:** 1 Department of Psychiatry, Graduate School of Medicine, Osaka University, D3 2-2 Yamada-oka, Suita, Osaka 565-0871 Japan; 2 Department of Psychiatry, Nippon Life Hospital, Osaka, Japan; 3 Department of Neuropsychiatry, Kochi University, Kochi, Japan; 4 UCM-UPM Centre for Biomedical Technology, Department of Cognitive and Computational Neuroscience, Complutense University of Madrid, Madrid, Spain; 5 The KEY Institute for Brain-Mind Research, University Hospital of Psychiatry, Zurich, Switzerland; 6 Department of Clinical Psychology and Psychobiology, La Laguna University, Tenerife, Spain; 7 Graduate School of Comprehensive Rehabilitation, Osaka Prefecture University, Osaka, Japan

**Keywords:** Psychiatric disorders, Neurophysiology

## Abstract

Idiopathic normal pressure hydrocephalus (iNPH) is a neuropsychiatric disease characterized by gait disturbance, cognitive deterioration and urinary incontinence associated with excessive accumulation of cerebrospinal fluid (CSF) in the brain ventricles. These symptoms, in particular gait disturbance, can be potentially improved by shunt operation in the early stage of the disease, and the intervention associates with a worse outcome when performed late during the course of the disease. Despite the variable outcome of shunt operation, noninvasive presurgical prediction methods of shunt response have not been established yet. In the present study, we used normalized power variance (NPV), a sensitive measure of the instability of cortical electrical activity, to analyze cortical electrical activity derived from EEG data using exact-low-resolution-electromagnetic-tomography (eLORETA) in 15 shunt responders and 19 non-responders. We found that shunt responders showed significantly higher NPV values at high-convexity areas in beta frequency band than non-responders. In addition, using this difference, we could discriminate shunt responders from non-responders with leave-one-subject-out cross-validation accuracy of 67.6% (23/34) [positive predictive value of 61.1% (11/18) and negative predictive value of 75.0% (12/16)]. Our findings indicate that eLORETA-NPV can be a useful tool for noninvasive prediction of clinical response to shunt operation in patients with iNPH.

## Introduction

Idiopathic normal pressure hydrocephalus (iNPH) is a neuropsychiatric disorder characterized by symptoms of gait disturbance, cognitive deterioration and urinary incontinence without any preceding diseases. Brain imaging by Magnetic Resonance Imaging (MRI) and Computed Tomography (CT) shows typical morphologic abnormalities such as ventriculomegaly, dilation of the Sylvian fissures and narrowing of the sulci and subarachnoid spaces over the high-convexity area of the brain, indicating excessive accumulation of cerebrospinal fluid (CSF) in the ventricles^1^. This morphological feature was defined as disproportionately enlarged subarachnoid-space hydrocephalus (DESH) in Japanese diagnostic criteria for iNPH^1^. The prevalence of possible iNPH was reported to be 1–2% in the elderly population by recent community or population-based studies^1–6^. However, many elderly patients with iNPH remain undiagnosed and untreated.

Unlike other types of dementia, iNPH has symptoms, especially gait disturbance, that can be potentially recovered with CSF shunt operation. Surgery is not effective in all cases, and when it is delayed there are less chances of getting a good clinical outcome^7–9^. Therefore, identifying predictive factors of shunt response is of considerable clinical importance. Japanese clinical guidelines for iNPH recommended “CSF tap test” by draining 30 ml of CSF by lumbar puncture to estimate possible shunt response based on symptoms recovery^1^. However, lumbar punction is an invasive procedure having some risks, including infection, bleeding and postural headache. In addition, it has the problem of low negative predictive value (18–50%) which means that patients that do not respond to the lumbar tapping (CSF tap negative patients) may show symptoms improvement if shunt operation is performed^10–12^. Several recent MRI studies found that presurgical morphological features of iNPH (e.g. high-convexity tightness, Sylvian fissure dilation and steep callosal angle) were related to a better shunt response^13–15^. However, prediction of shunt response based only on a morphological feature of DESH also suffers from a low negative predictive value (25%)^16^. Some studies using Single Photon Emission Computed Tomography (SPECT) and Positron Emission Tomography (PET) reported that shunt responders showed significant lower cerebral blood flow in the basal frontal lobes compared to non-responders^17,18^. Most PET, SPECT or functional MRI (fMRI) studies, however, have failed to detect presurgical difference in cerebral blood flow between shunt responders and non-responders before shunt operation^1^.

Electroencephalography (EEG) is a noninvasive and widely available tool commonly used in neuroscience research to investigate cortical electrical activities. It is also used in clinical practice to support diagnoses of epilepsy, disturbance of consciousness and dementia. Unlike PET, SPECT and fMRI which measure glucose metabolism or blood flow changes secondary to cortical activities, EEG directly measures electrical potentials with high temporal resolution (1–2 ms). This activity can be obtained through electrodes placed on the scalp, representing a linear mixture of cortical electrical potentials from different brain sources. Thus, to visualize the cortical electrical activity from EEG data, it is necessary to solve a linear inverse problem of EEG. Exact-low-resolution-brain-electromagnetic-tomography (eLORETA) is a linear inverse solution that reconstructs cortical electrical activity from EEG data with correct localization^19–23^. eLORETA with EEG data has been widely used in neuroscience studies^20,24–28^. However, traditional EEG power spectral analysis and eLORETA analysis have failed to discriminate shunt responders from non-responders^29^. In a previous study using EEG and normalized power variance (NPV) analysis, a sensitive measure of the instability of cortical electrical activity^19,30^, we showed that shunt responders had significantly higher NPV at the right fronto-temporo-occipital electrodes (Fp2, T4 and O2) in beta frequency band compared to non-responders^29^.

NPV is theoretically thought to sensitively reflect the instability of cortical electrical activity related to phase transition^30^. Using NPV analysis with EEG data in epilepsy, we demonstrated the high sensitivity of this method to visualize the instability of cortical electrical activity at the seizure onset zone in the pre-ictal phase and its stabilization after transition to the ictal phase^19^. In this study, we could assume a phase transition from instability of cortical electrical activity in shunt responders to stabilization in non-responders because pathophysiological changes in these patients are in reversible and irreversible to shunt operation, respectively^30^. Thus, we decided to apply NPV analysis with eLORETA cortical electrical activity (eLORETA-NPV analysis), as it is able to detect differences in phase-instability of cortical electrical activity between shunt responders and non-responders.

In the present study, using eLORETA-NPV analysis, we aimed to detect differences in cortical electrical oscillations between shunt responders and non-responders and to determine cortical regions responsible for shunt response. This would provide valuable information for improving prediction of shunt response and understanding the neurophysiological mechanisms of symptom recoveries induced by shunt operation in iNPH.

## Results

### Demographic and clinical results

We classified 34 iNPH patients into 15 shunt responders and 19 non-responders based on shunt operation outcome, as described in the Methods section. Most of the patients in the shunt responder group showed improvement only in the gait domain, except for a patient who showed improvement in both gait and cognitive domains and another patient who showed improvement only in the cognitive domain. Demographic and clinical characteristics of our subjects are shown in Table 1. There were no significant group differences for age, gender, and clinical scores. The rate of shunt responders was relatively low because our classification criteria of shunt response were more stringent than those of the Japanese Clinical Guidelines in order to select patients who demonstrated significant improvements in gait and cognitive symptoms as shunt responders^31^.

**Table 1:** Cognitive and gait function test scores of Shunt responders and Non-responders.

Test	Shunt responders	Non-responders	p-value
Gender (F/M)	6/9	8/11	0.90
Age	75.0 ± 6.6	76.0 ± 6.0	0.93
WT	27.5 ± 19.6	21.8 ± 6.3	0.32
TUG	15.8 ± 5.8	13.7 ± 4.9	0.26
GSSR	6.9 ± 3.2	6.0 ± 2.0	0.34
MMSE	23.2 ± 3.7	24.4 ± 3.9	0.34
FAB	11.1 ± 2.7	11.8 ± 2.6	0.42
TMT-A	119 ± 78	105 ± 90	0.65
WMS-R_Attention/Concentration index	80.0 ± 15.2	85.7 ± 12.1	0.24
WAIS-III-Digit Symbol-Coding	5.8 ± 2.7	7.2 ± 2.6	0.14
WAIS-III-Block Design	6.5 ± 3.0	7.3 ± 2.1	0.43

Data are mean ± SD. Difference in the female to male ratios of shunt responders and non-responders is assessed using Chi-square test. Differences in the other scores are assessed using paired Student’s t test (uncorrected). WT: 10-m reciprocating walking test, TUG: 3 m Timed Up and Go, GSSR: Gait Status Scale-Revised, MMSE: Mini-Mental State Examination, FAB: Frontal Assessment Battery, TMT-A: Trail Making Test Part A, WMS-R: Wechsler Memory Scale-Revised, WAIS-III: Wechsler Adult Intelligence Scale-III.

### eLORETA analysis results

There was no significant difference in cortical electrical activities between shunt responders and non-responders, as indicated by eLORETA analysis.

### eLORETA-NPV analysis results

**Figure 1:**
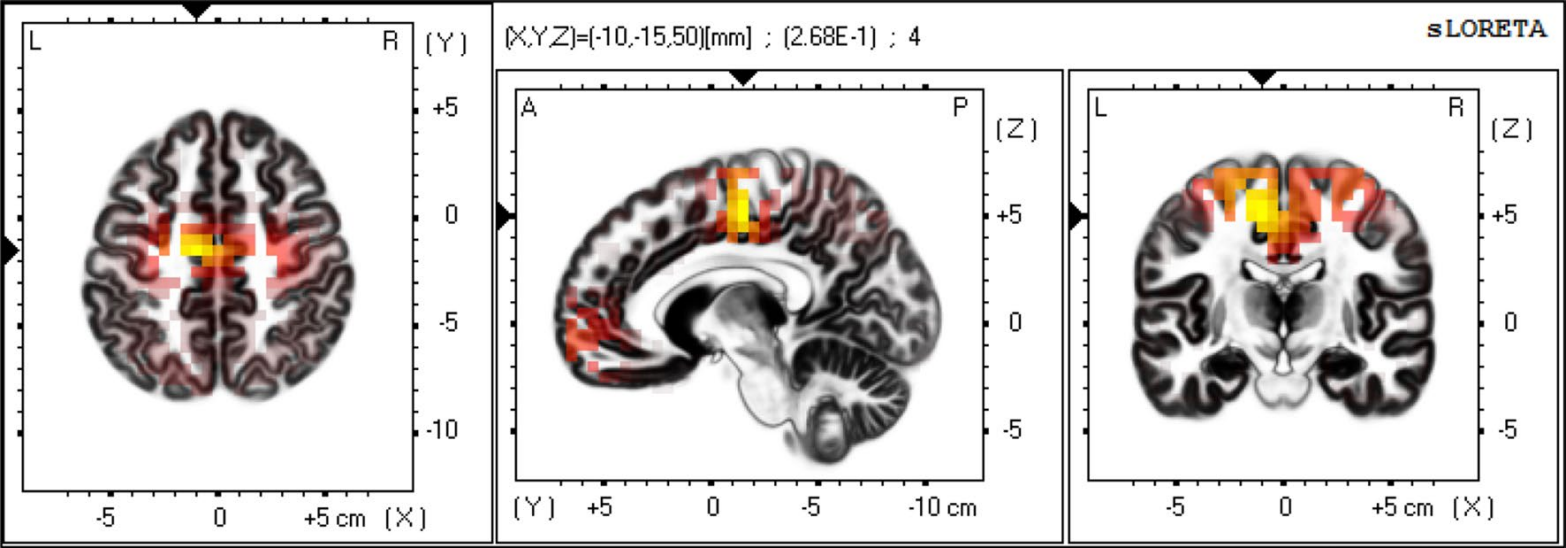
eLORETA-NPV in beta frequency band of Shunt responders. Mean eLORETA-NPV value in beta frequency band of 15 Shunt responders. Normalized power variance (NPV) of eLORETA cortical electrical activity is color coded from grey (zero) to red to bright yellow (maximum). Slices from left to right are axial, sagittal and coronal images (viewed from top, left and back). L, left; R, right; A, anterior; P, posterior.

**Figure 2:**
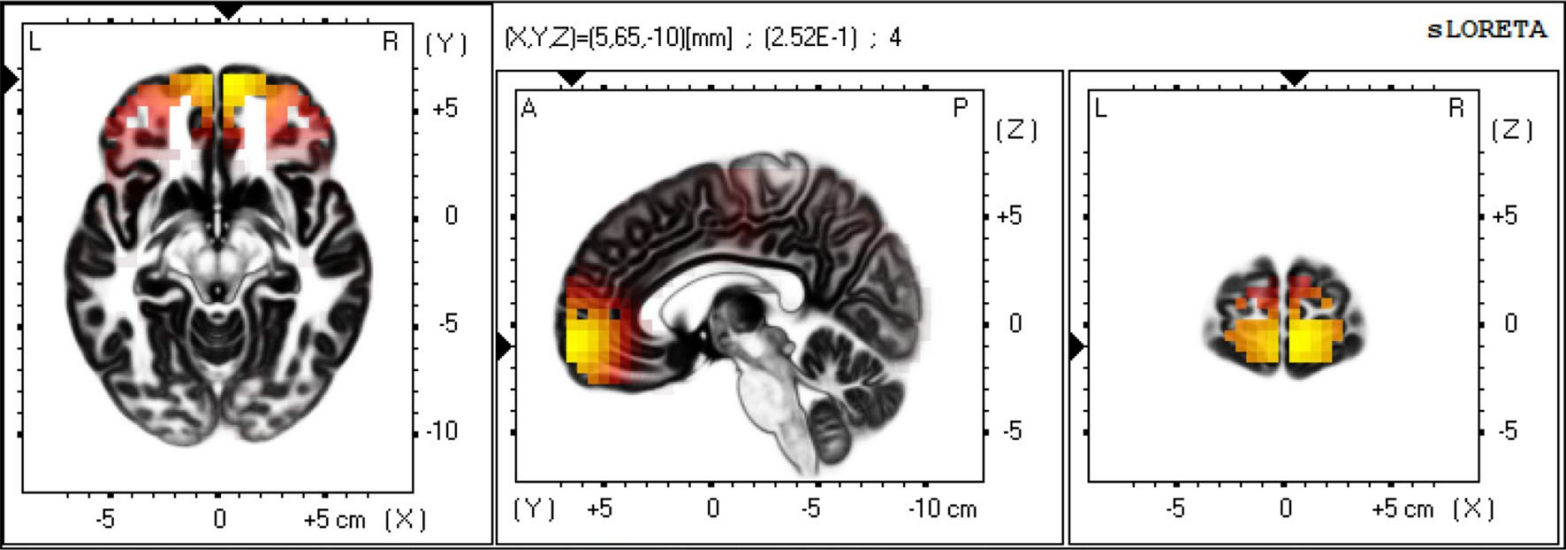
eLORETA-NPV in beta frequency band of Non-responders. Mean eLORETA-NPV value in beta frequency band of 19 Non-responders. Normalized power variance (NPV) of eLORETA cortical electrical activity is color coded from grey (zero) to red to bright yellow (maximum). Slices from left to right are axial, sagittal and coronal images (viewed from top, left and back). L, left; R, right; A, anterior; P, posterior.

**Figure 3:**
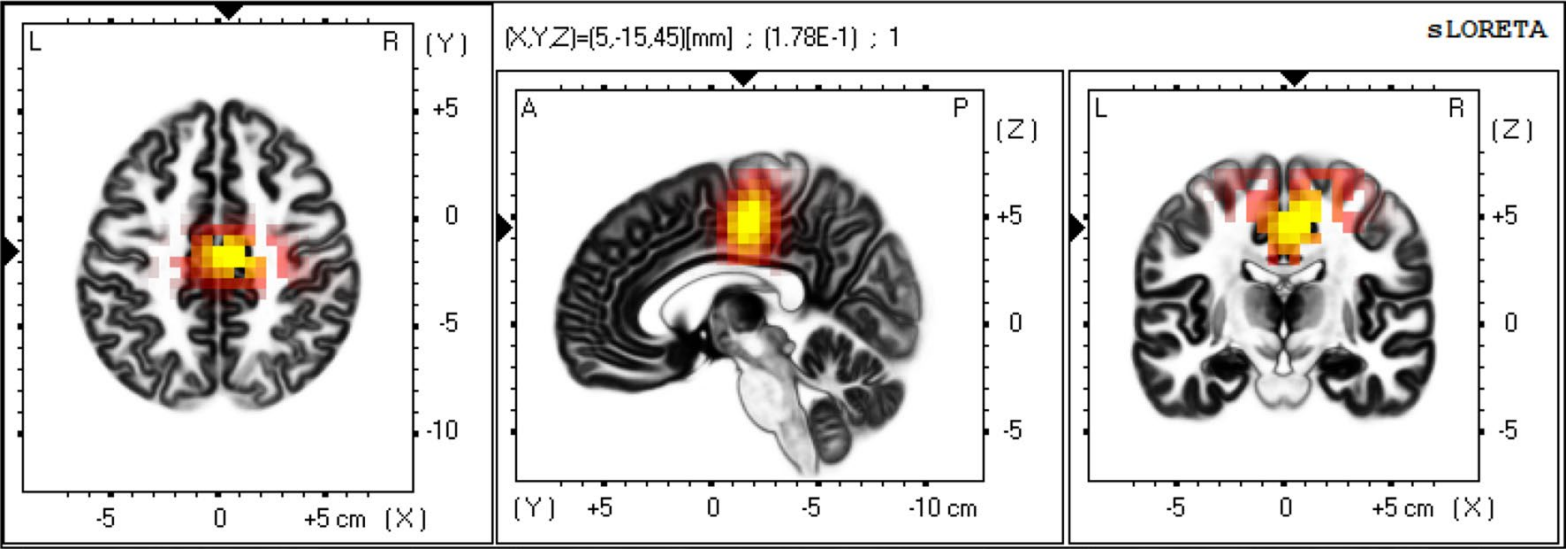
Log of F-ratio of eLORETA-NPV in beta frequency band between Shunt responders and Non-responders. Shunt responders had significantly higher normalized power variance (NPV) of eLORETA cortical electrical activity in beta frequency band at the high-convexity area (cingulate gyrus, paracentral lobule and medial frontal gyrus) compared to Non-responders. Log of F-ratio of eLORETA-NPV values in beta frequency band between 15 shunt responders and 19 non-responders above threshold (p < 0.05) is color coded red to bright yellow (maximum). Slices from left to right are axial, sagittal and coronal images (viewed from top, left and back). L, left; R, right; A, anterior; P, posterior.

Shunt responders showed significantly higher eLORETA-NPV values at high-convexity areas (i.e. cingulate gyrus, paracentral lobule and medial frontal gyrus) in beta frequency band relative to non-responders as shown in Figs. 1, 2, 3 [extreme p = 0.044 at the cingulate gyrus (X = 5, Y =  − 15, Z = 45; MNI coordinates)]. Using this beta eLORETA-NPVs at the cingulate gyrus, discriminant function analysis yielded a discriminant function that predicts shunt response. The function is as follows:

$$\text{Prediction score}={\log}(\text{beta eLORETA-NPV})+1.49$$

where positive and negative scores indicate positive and negative shunt response respectively. This index could correctly discriminate shunt responders from non-responders with leave-one-subject-out cross-validation accuracy of 67.6% (23/34) [positive predictive value of 61.1% (11/18) and negative predictive value of 75.0% (12/16)].

## Discussion

In this study, we employed eLORETA-NPV analysis, a sensitive measure of the instability of cortical electrical activity, to determine cortical regions related to shunt response and to find EEG predictive markers of shunt response in patients with iNPH. Our main findings were that: (1) shunt responders had significantly higher eLORETA-NPV values at high-convexity areas in beta frequency band relative to non-responder (Figs. 1, 2, 3), and (2) using this difference in eLORETA-NPV values, we could correctly discriminate shunt responders from non-responders with a positive predictive value of 61.1% (11/18) and a negative predictive value of 75.0% (12/16) with cross-validation.

The high-convexity areas found associated with shunt response in our study are regions considered to be compressed by dilated ventricles in iNPH. The reduction of this compression might be associated with the pathophysiological mechanisms of symptoms recovery induced by shunt operation^1,13–15^. In support to our results, several recent studies using MRI showed that presurgical compression of high-convexity areas was a significant predictor of better shunt operation outcome^13–15^. A recent large-scale MRI study reported lack of correlation between presurgical MRI morphological features and postsurgical outcome^32^. In that study, the majority of iNPH patients did not undergo CSF tap test. Most SPECT, PET and fMRI studies have failed to detect difference in cerebral blood flow between shunt responders and non-responders prior to shunt operation^1^. Similarly, traditional EEG power spectral analysis and eLORETA analysis have failed to discriminate shunt responders from non-responders^29^. Unlike these neuroimaging modalities, eLORETA-NPV analysis with EEG data could detect difference of cortical electrical activities related to shunt response at high-convexity areas. To date, for prediction of shunt response CSF tap test has been the method used in clinical practice in iNPH. However, as previously mentioned, CSF tapping is an invasive procedure, and has several risks (e.g. infection, bleeding and post-punction headache). Our finding suggests that eLORETA-NPV value at the high-convexity areas in beta frequency band can be a noninvasive predictive marker of shunt response without the need of performing a lumbar puncture to assess symptoms improvement.

It is noteworthy that the difference in cortical electrical activity between shunt responders and non-responders in this study was found in beta frequency band. Responders mainly improved in gait disturbance. This result of frequency band seems to be in line with the role of beta oscillations in motor control. Furthermore, abnormalities in this frequency band have been closely associated with motor disorders^33,34^. Interestingly, in our previous study using NPV analysis with EEG data, differences in EEG oscillations in iNPH patients were also found in the beta band^29^. Consistent with the source location finding at the right convexity areas in this study, we have provided evidence using eLORETA-ICA indicating that the right ventral attention network was involved in gait speed recovery after CSF drainage^27^. Also, in support to our findings, recent neuroimaging and electrophysiological studies revealed that the cortical locomotor network consists of dorsal and ventral pathways. Interestingly, the dorsal and ventral locomotor networks encompass areas of the paracentral sensorimotor cortex and ventral attention network regions, respectively^35,36^.

In this study, we assumed a functional transition from reversible phase to irreversible phase in iNPH (i.e. from shunt responder to non-responder). Theoretically, Thermal and statistical physics suggests that a reversible phase is unstable and irreversible phase is a stable state, showing high and low variances of state parameters, respectively^30^. This supports our result of shunt responders showed significantly higher eLORETA-NPV values compared to non-responders.

This is the first study demonstrating that, unlike other methods such as SPECT, PET, fMRI, and EEG with eLORETA power analysis that have been used to investigate brain activity, EEG with eLORETA-NPV analysis can detect differences in cortical electrical oscillations between shunt responders and non-responders and localize the brain sources associated with shunt operation outcome in iNPH patients. This may be attributed to the fact that EEG directly detects cortical electrical activity with high temporal resolution (milliseconds), eLORETA analysis reconstructs cortical electrical activity from EEG data with correct localization and that NPV analysis sensitively detect instability of cortical electrical activity. Overall findings indicate that eLORETA-NPV is a useful and sensitive tool to assess cortical states and can provide valuable information for understanding the neurophysiological mechanisms underlying this disease.

Our results should be interpreted with caution based on the following limitations. First, the sample size of iNPH patients was relatively small. Therefore, our findings should be considered as preliminary, and replicated in larger samples. Nevertheless, our main results of eLORETA-NPV difference between shunt responders and non-responders are in line with previous neuroimaging findings of iNPH. This allows us to suggest that eLORETA-NPV is a reliable measure of instability of cortical electrical activity. Second, patients with iNPH have atrophy and deformation of the brain cortex relative to the realistic head model used in eLORETA-NPV. However, we found difference in eLORETA-NPV values between shunt responders and non-responders among several lobules at the high-convexity area. This may indicate that our results are robust to cortical atrophy and deformation effect. Third, results of analysis of variance (ANOVA) were uncorrected for the five tests performed on eLORETA-NPV data for each frequency band. This was because eLORETA-NPV values of each frequency band followed the chi-squared distribution of different degrees of freedom. Thus, we could not apply the permutation test implemented in eLORETA Statistics to frequency domain of eLORETA-NPV data to correct for multiple comparisons^37^. However, our eLORETA-NPV results of beta frequency band and cortical regions discriminating shunt responders from non-responders were consistent with findings from previous neuroimaging studies of iNPH^13–15,29^.

Overall, a finding with important therapeutic implications of our study is that eLORETA-NPV analysis can be used for prediction of response to shunt operation in patients with iNPH. It is promising that eLORETA-NPV may also be useful to assess or predict brain states in patients with other neuropsychiatric disorders.

## Methods

### Subjects

Definite and probable iNPH patients who underwent shunt operation at the Department of Neurosurgery of Osaka University Hospital between April 2010 and May 2019 were consecutively recruited from the Neuropsychology Clinic at the Department of Psychiatry of Osaka University Hospital. CSF tap test was performed on all patients. Then, patients who showed positive response underwent shunt operation according to the Japanese Clinical Guidelines for iNPH^1^.

The inclusion criteria were: (1) age 60 years or older; (2) at least one of the triad symptoms: gait disturbance, cognitive impairment and urinary incontinence; (3) dilated cerebral ventricles and narrowed CSF space at the high convexity areas without severe cortical atrophy on MRI; (4) absence of diseases or conditions that might cause clinical symptoms and neuroimaging findings; (5) no history of severe head trauma, subarachnoid hemorrhage, meningitis and tumor; (6) normal CSF pressure (< 200 mm H_2_O) and contents by lumbar puncture and (7) right handedness. Exclusion criteria were: (1) comorbidities of motor or psychiatric disorders except for Alzheimer’s disease (AD) (2) long-standing overt ventriculomegaly in adults (LOVA) on MRI findings and (3) a lack of artifact free epochs of 500-s (500-s) in the EEG recordings. We included iNPH patients with AD comorbidity as AD is commonly seen in iNPH and AD pathology is considered not to affect clinical recovery after shunt operation^38,39^.

Out of 43 patients who met the inclusion criteria, two patients were excluded due to comorbidity of Parkinson’s disease, two patients due to LOVA on MRI findings, four patients due to lack of 500-s artifact free epochs, and one patient that did not complete followed up after shunt operation. Finally, 34 patients were included in this study.

This study was approved by the Ethics Committee of Osaka University Hospital and written informed consent was obtained from the patients or their families. CSF tap test and shunt operation were performed in accordance with the Japanese Clinical Guidelines for iNPH^1^.

### Gait assessment

Assessments of gait and cognition have already been described in our previous study^31^. Briefly, gait disturbance was assessed by the 10-m reciprocating walking test (WT), the 3 m Timed Up and Go (TUG) test^40^ and the Gait Status Scale-Revised (GSSR)^41^. The thresholds of improvement were set at 10% improvement in the WT and TUG, and 1-point improvement in the GSSR. Improvement in all gait assessments was defined as clinical improvement in the gait domain.

### Cognitive assessment

Cognitive impairment was assessed by the Frontal Assessment Battery (FAB)^42^, Mini-Mental State Examination (MMSE)^43^, Wechsler Memory Scale-Revised (WMS-R)-Attention/Concentration Index^44^, Wechsler Adult Intelligence Scale-III (WAIS-III)-Block Design^45^, WAIS-III-Digit Symbol Coding^45^, and Trail Making Test Part A (TMT-A)^46^. The improvement thresholds of the cognitive tests were set at 2, 3, 15, 3, 3 points and 30% respectively. Improvement in more than half of the cognitive assessments was defined as clinical improvement in the cognitive domain.

### Assessment of shunt response

Assessment of shunt response has already been described in our previous study^29^. Briefly, before and after shunt operation, gait disturbance and cognitive impairment were evaluated at presurgical evaluation and postsurgical evaluations at 1, 3, 6 months, and 1 year. Urinary incontinence was excluded from assessment in our study because of low reliability, as the frequency of urination was sometimes self-reported. Shunt operation was judged as positive if at least one symptom showed clinical improvement at any time of assessment after shunt operation; otherwise it was judged as negative. Consequently, the patients were classified as shunt responders and non-responders. These classification criteria were more stringent than those of the Japanese Clinical Guidelines for iNPH^1^, because we selected patients who showed significant symptoms recovery as shunt responders^31^.

### EEG recording

EEG data acquisition and the procedure of eLORETA have already been described in detail in our previous studies^20,31^. EEG data was recorded 1–3 month before shunt operation during eyes-closed resting-state condition for about 20 min using a 19-electrode EEG system (EEG-1000/EEG-1200, Nihon Kohden Inc., Tokyo, Japan), and filtered through a band-pass filter of 0.53 to 120 Hz with a sampling rate of 500 Hz. Subjects were instructed to relax but remain awake. During the EEG sessions, drowsiness was avoided by giving instructions once again. For each patient, 500-s artifact free epochs were selected off-line, and imported into eLORETA and eLORETA-NPV analysis.

### eLORETA analysis

Here is a brief description. eLORETA is a linear weighted minimum norm inverse solution which has the property of correct localization albeit with low spatial resolution^22,23^. The solution space consists of 6,239 voxels in the cortical grey matter at 5 mm spatial resolution, in a realistic head model^47^ with the MNI152 template^48^. The eLORETA method has been widely used to explore cortical electrical activities and its validity has been proved with real human data during various sensory stimulations and in neuropsychiatric disease studies^19–21,49^. In this study, eLORETA and eLORETA-NPV analyses were performed in MATLAB R2019b software. In eLORETA analysis, first, 500-s EEG data of each subject were transformed into the time–frequency domain after discrete Fourier transform using ft_freqanalysis function in Field Trip toolbox (https://www.fieldtriptoolbox.org/) installed in MATLAB software. Then, the time–frequency data was transformed to the cortical electrical current density data by multiplying spatial filter of eLORETA which was obtained using MATLAB program written by author R. B. This program followed a technical instruction about eLORETA provided by author R. D. P.-M.^22^ and we confirmed equality of the obtained spatial filter with that of eLORETA. Finally, the cortical electrical activity was obtained by squaring the cortical electrical current density. The eLORETA analysis was computed for five frequency bands: delta (1.5–4.0 Hz), theta (4.5–7.0 Hz), alpha (7.5–13.0 Hz), beta (13.5–29.5 Hz), and gamma (30.0–59.5 Hz). Before statistical comparison, to compare across different subjects, the subject-wise normalization implemented in eLORETA Statistics was applied to the data.

### eLORETA-NPV analysis

In eLORETA-NPV analysis program, the NPV value of cortical electrical activity was calculated at each cortical voxel for each frequency band, with eLORETA-NPV value being defined as the variance of cortical electrical activity divided by the square of mean cortical electrical activity for each 4.6-s EEG epoch. Then, eLORETA-NPVs of 4.6-s EEG epochs were collected at 1.15-s steps for the whole EEG epoch and averaged to get the stationary mean eLORETA-NPV value (moving average filter method)^31^. The eLORETA-NPV analysis was computed for five frequency bands: delta (1.5–4.0 Hz), theta (4.5–7.0 Hz), alpha (7.5–13.0 Hz), beta (13.5–29.5 Hz), and gamma (30.0–59.5 Hz).

### Statistical group analysis and discriminant function analysis

The differences in eLORETA-NPV values at each cortical voxel in each frequency band between shunt responders and non-responders were assessed using ANOVA implemented in eLORETA Statistics. The level of significance for this test was set at p < 0.05 with correction for multiple comparisons across 6,239 cortical voxels using a permutation test^37^. The eLORETA-NPV values of each frequency band followed the chi-squared distribution of different degrees of freedom, thus we applied the permutation test correction in eLORETA Statistics to the 6,239 cortical voxels but not to five frequency bands. In order to find an prediction score for shunt response, we selected eLORETA-NPV values at the cortical source (cingulate gyrus: X = 5, Y = − 15, Z = 45) in the specific frequency band (beta frequency) that showed most significant differences between the two groups as a predictor and performed discriminant function analysis using the SPSS software version 25.0 (SPSS Japan Inc., and IBM Company Tokyo, Japan).

## Data Availability

The datasets generated during and/or analyzed during the current study are available from the corresponding author on reasonable request.
